# POOR ACTIVITIES OF DAILY LIVING PREDICT FUTURE WEIGHT LOSS IN OLDER ADULTS AFTER HOSPITAL DISCHARGE

**DOI:** 10.1093/geroni/igad104.2463

**Published:** 2023-12-21

**Authors:** Alfons Ramel

**Affiliations:** University of Iceland, Reykjavik, Hofuoborgarsvaoio, Iceland

## Abstract

The aim was to investigate whether older adults with poor activities of daily living (ADL) at hospital discharge had increased weight loss after six months of follow-up and whether nutrition therapy can prevent this weight loss. This was a secondary analysis of the HOMEFOOD dietary intervention trial (N=104) investigating community dwelling older adults (66-95 years) discharged from hospital and at risk for malnutrition, receiving either 6 months nutrition therapy or standard care without any further nutrition intervention. ADL were assessed using seven questions on self-care including mobility.Of the participants, 45 (43%) had good ADL at discharge, 36 (35%) had medium ADL and 23 (22%) had poor ADL with no differences between control and intervention group. During the study period, 79% of the control group lost more than 1 kg body weight with a mean weight loss of -3.5 ± 3.9 kg, whereas only 2% of the intervention group lost more than 1 kg with a mean weight gain of 1.7 kg ± 2.5 kg. According to general linear models, participants in the control roup with poor ADL had significantly higher weight loss than participants with good ADL (estimated and adjusted: 3.6 kg (95%CI: 1.0-6.1 kg, P=0.007). No such difference was observed in the intervention group. In our study, participants with poor ADL at hospital discharge develop lower body weight by around 3.5 kg six months later when compared to participants with good ADL. Receiving nutrition therapy post discharge can nullify these differences in body weight between ADL categories.

